# Two Cases of Simple Fracture of the Skull Unrecognized for Days—Late Appearance of Symptoms—Trephining—Results

**Published:** 1882-10

**Authors:** J. H. Pryor

**Affiliations:** Resident Physician Buffalo General Hospital


### Clinical Reports.

### Two Cases of Simple Fracture of the Skull Unrecognized for Days—Late Appearance of Symptoms. Trephining—Results.

Reported by J. H. Pryor, M. D.,
Resident Physician Buffalo General Hospital.

C. S., æt. 23, S. Ireland, sailor, entered hospital May 25, 1881. Two hours before, he had fallen through a hole in a dry-dock to the floor beneath, a distance of seventeen feet, sustaining a colle's fracture, and a slight bruise over the right eye. No signs or symptoms of fractured skull. The colle's fracture was dressed and he was put in bed, complaining of nothing more than a slight headache. He was given his clothes after a day or two, and allowed to sit up. June 5th, he spoke of being dizzy at times. June 7th, the pupils were noticed to be of unequal size, one remaining persistently dilated. June 11th, he could not read because the printed words ran together and he saw everything in pairs; also, had a slight convergent strabismus, and was more stupid than before. Thinking it probable that a simple fracture had occurred, Dr. Gay, the visiting surgeon, cut down where the contusion had been, as evidence pointed toward that spot. Upon lifting the flap a star-shaped fracture was disclosed with depression. The skull was trephined and the depressed bone elevated. A large clot lay upon the dura mater, but it was not removed. Allowance for drainage was made. After three days he grew brighter and the pupils assumed their normal shape. July 19th, he was discharged, well. The healing of the wound was delayed because the pus infiltrated the tissues of the upper eyelid, causing a troublesome complication.

*Case No. II.*—M. B., æt. 23, S. Poland, entered hospital Aug. 16, 1882. Patient walked into the hospital unaided. A nurse assigned him a chair and he sat down with his hand pressed to his forehead. When asked why he had come, he replied, because he had a headache; that he had fallen into an excavation where sewer pipes were being laid; that for a time he was covered with earth which had become detached from the sides, and he thought that something had struck upon his head. A careful examination of the scalp was made and naught but a slight abrasion over the occipital region was noticed. I pressed upon it; could feel no depression, and it caused him no pain. There were no symptoms of concussion or compression, only a headache. Now during the last few months several patients have found their way into the hospital who had fallen into a hole and had either inspired street gas or had not inspired any air at all for a time, and these men acted much as this man did; they recovered in a night and a day, helped by sleep and rest; he was put in bed and given pot. brom. gr. xxx. Soon he was asleep, breathing naturally. On the third morning after entrance his only complaint was of a headache in the frontal region; temperature and pulse normal. On the fourth morning, however, while making my rounds, I offered him my hand and found, to my surprise, that he could neither reach nor grasp it, dolefully explaining that he had lost the power of moving his arm during the night. Nor could he move his leg, yet he had sensation in both. I immediately bethought me of the abrasion I had noticed. Finding the spot, I made pressure over it, when he gave a cry of pain. Believing that a fracture existed which had not been recognized on account of the paucity of symptoms, I communicated with Dr. W. C. Phelps, the attending surgeon. He decided to make an exploratory incision. A diagnosis of fracture was made, and after a good-sized flap was raised, there was revealed a fracture with depression at the junction of the lambdoid and sagittal sutures, which presented the appearance that two capital Y's would, if their stems were joined, and implicating the occipital and both parietal bones. It became necessary to trephine three times before the depressed bone could be elevated. A large clot was found and removed, also a spiculum of bone which had been driven through the membranes of the brain and penetrated the brain substance. Eight hours after the operation the patient could move his arm. The next day he could move his foot a little. Mental condition not much changed. Same night temperature 104°, pulse 120. Next day 105°; at night, 106½°, pulse 132. From this time the temperature and pulse dropped, and he gradually improved. Aug. 28th, was better, no pain; appetite fair; temperature 100°, pulse 90. Aug. 30th, he developed facial erysipelas, was sent to the pavilion and came under Dr. Cary's care, the visiting physician. While suffering from erysipelas, swelling of the parotids was noted. His temperature ran very high, usually 106°. Sept. 3d, pronounced convalescent from erysipelas, suffering from pyæmia. Sept. 5th, temperature, at midnight, 107½°, pulse 140. Same day died at 4 o'clock, P. M. Sept. 6th, autopsy. Pericranium stained with blood. Inside of scalp greatly congested. Fracture of skull from the nasal notch to the foramen magnum. Frontal bone fractured at the line of suture. Sagittal suture separated, at the junction of the lambdoid and sagittal sutures, the lines of fracture extended in many directions, considerable depression of the inner plate with none of the outer. Occipital bone fractured from this point to the foramen magnum, the line of fracture tending to the right enough to avoid the occipital protuberance. Dura mater reddened and marked with pus and lymph. Where the spicula of bone had entered, the brain tissue had undergone a degenerative change. There was a large clot near this point. No effusion.

